# Efficacy and Moderation of Mobile App–Based Programs for Mindfulness-Based Training, Self-Compassion Training, and Cognitive Behavioral Psychoeducation on Mental Health: Randomized Controlled Noninferiority Trial

**DOI:** 10.2196/mental.8597

**Published:** 2018-10-11

**Authors:** Winnie WS Mak, Alan CY Tong, Sindy YC Yip, Wacy WS Lui, Floria HN Chio, Amy TY Chan, Celia CY Wong

**Affiliations:** 1 Diversity and Well-being Laboratory Department of Psychology The Chinese University of Hong Kong Shatin, NT China (Hong Kong); 2 Center for Personal Growth and Crisis Intervention of the Corporate Clinical Psychology Services Hospital Authority Hong Kong China (Hong Kong); 3 Department of Counselling and Psychology Hong Kong Shue Yan University Hong Kong China (Hong Kong); 4 Department of Psychology University of Houston Houston, TX United States

**Keywords:** mental health, mobile apps, mindfulness, compassion

## Abstract

**Background:**

Mindfulness-based interventions, self-compassion training, and cognitive behavioral therapy have garnered much evidence in its salutary effects on mental health. With increasing application of smartphone and mobile technology on health promotion, this study investigated the efficacy and possible moderators of mindfulness, self-compassion, and cognitive behavioral psychoeducation training mobile apps in the improvement of mental health.

**Objective:**

The aim of this study was to examine the efficacy of 3 mobile app–based programs: mindfulness-based program, self-compassion program, and cognitive behavioral psychoeducation program in improving mental well-being and reducing psychological distress. Changes in mindful awareness and self-compassion were also assessed. To further delineate the suitability of each program for different types of individuals, individual difference variables (ie, discomfort with emotions and tolerance for ambiguity) were explored for potential moderation.

**Methods:**

This study was a 3-arm, randomized, controlled, noninferiority trial examining the efficacy of mindfulness-based program, self-compassion program, and cognitive behavioral psychoeducation. Participants were randomized into either 1 of the 3 conditions. Throughout the 4-week, 28-session program, participants spent 10-15 min daily reviewing the course content and practicing various related exercises. At preprogram, postprogram, and 3-month follow-up, participants also completed Web-based measures of mental well-being, psychological distress, mindful-awareness, and self-compassion as well as the proposed moderators.

**Results:**

Among the 2161 study participants, 508 and 349 completed the post- and 3-month follow-up assessment, respectively. All 3 conditions (mindfulness-based program: N=703; cognitive behavioral psychoeducation: N=753; self-compassion program: N=705) were found to be efficacious in improving mental well-being and reducing psychological distress. All conditions enhanced mindful awareness at postprogram. Significant interaction effect was found on self-compassion; cognitive behavioral psychoeducation and self-compassion program, but not mindfulness-based program, significantly enhanced self-compassion at postprogram. No significant differences regarding usage and users’ satisfaction were found among the 3 conditions. None of the proposed moderators were found to be significant.

**Conclusions:**

Mindfulness-based, self-compassion, and cognitive behavioral psychoeducation mobile apps were efficacious in improving mental well-being and reducing psychological distress among adults at postprogram and 3-month follow-up. Future app-based psychological training programs should consider gamification and personalization of content or feedback to enhance engagement and mitigate the high attrition rates that are common in app-based health promotion programs.

**Trial Registration:**

Chinese Clinical Trial Registry (ChiCTR) ChiCTR-TRC-13003468; http://www.chictr.org.cn/hvshowproject.aspx?id=6220 (Archived by WebCite at http://www.webcitation.org/734PlOz50)

## Introduction

### Mobile Mental Health

Mental health is an essential part of health that contributes to individuals’ overall well-being [1]. However, about 450 million people suffer from mental or behavioral disorders worldwide [2]. According to a recent territory-wide epidemiological study conducted in Hong Kong, the prevalence rate of common mental disorders in Hong Kong was estimated to be around 13.3%, with the highest prevalence among adults aged 26 to 35 years [3]. Furthermore, only 26% of these individuals sought mental health services in the past year. Given that mental ill health causes tremendous burden to individuals, families, and society, mental health promotion should be advocated and propagated in the community.

With the increasing utilization of mobile phones and tablet devices, mobile intervention becomes a viable option to educate individuals about mental health and to promote well-being. In Hong Kong, the number of mobile service subscribers was 16.72 million as of March 2016 [4], compared with 8 million in June 2012. The penetration rate of 228.3% in Hong Kong was one of the highest figures globally. The amount of mobile data usage has increased ten folds from 2006 to June 2016, demonstrating the rapid and continual increase of smartphone and internet usage [5].

Mobile apps have dominated the browsing time of mobile phone users. In a survey conducted by comScore, Inc. in 2012 [6], 82% of the time spent on mobile media happened via apps, and this percentage has risen to 90% in 2015 [7]. Given the ubiquitous nature of apps, such media can potentially provide a highly accessible, convenient, and anonymous way to promote mental health on a large scale to populations who would otherwise not seek help due to inconvenience, stigma, and other help-seeking barriers [8,9].

### Cognitive Behavioral–Based Psychoeducation Training

The cognitive behavioral approach has been widely applied and suggested to be one of the most evidence-based approaches in reducing psychological distress and promoting mental well-being [10]. In recent years, internet-based and mobile apps that are based on the cognitive behavioral approach have been developed to help people cope with stress, increase emotional awareness, and promote wellness [11-14]. The cognitive behavioral approach can modify ones’ emotion regulation, reduce psychological distress, and promote mental health by changing the cognitive appraisal process and the mood-related behaviors of the individuals [15]. Recently, Rathbone et al [16] reviewed 8 studies concerning the efficacy of cognitive behavioral therapy (CBT)-related mobile apps and concluded that these apps appeared to repeatedly show improvements in symptom severity on a range of psychological issues including depression and stress. Although the long-term effectiveness was unclear, the short-term effect was evident.

Cognitive behavioral approach is also culturally appropriate in the Chinese culture. The Chinese socialization process emphasizes on structure and hierarchy, clearly defined roles, and responsibility. As such, Chinese clients generally display low tolerance for ambiguity. Given that cognitive behavioral approach is directive and structured, it is suitable for the Chinese population [17]. In addition, as the Chinese culture is strongly influenced by Confucianism that emphasizes on the importance of education and learning, the psychoeducational component in CBT is especially suitable for the Chinese population. Chinese culture has a common belief that any desired change could be brought about by diligent learning. In Hwang’s [18] recommendation, to meet the therapeutic needs of Chinese clients, 1 principle is to promote psychoeducation that engages clients in their familiar *student* role. Mobile apps are well positioned to deliver cognitive behavioral–based psychoeducation as they can engage users with multimedia tools and provide clear information to aid understanding of mental health concepts [19].

### Mindfulness-Based Training

In addition to utilizing the cognitive behavioral approach to reduce stress and promote mental health, in the recent decade, ample research has demonstrated the power of mindfulness and self-compassion in promoting mental health [20-22]. These approaches have their theoretical roots in Asian philosophies, and they are culturally adaptive approaches among Chinese communities [23].

Mindfulness-based training is an approach that focuses on the cultivation of conscious awareness in the unfolding of events in the present moment [24]. It emphasizes the transience of all thoughts and feelings. It involves self-regulation of attention and orientation toward the present moment with openness [25]. Meta-analysis showed mindfulness-based training to have a medium effect size in improving anxiety (Hedges *g*=0.63) and depressive symptoms (Hedges *g*=0.59) across all samples. Mindfulness-based training has also been found to have a medium effect size (Hedges *g*=0.53) in comparison with waitlist control across a range of psychological issues, particularly stress, anxiety, and depression [26].

Mindfulness-based training is increasingly being delivered online because of technology advancement in recent years. Research on an 8-week internet-based mindfulness-based training showed that compared with waitlist control, internet-based mindfulness training improved university students’ and staffs’ mental well-being, and the effect was sustained at the 3-month follow-up [27]. Another study compared internet-based mindfulness training with internet-based cognitive behavioral training among college students and working adults and found that both were efficacious in improving mental health, psychological distress, life satisfaction, sleep disturbance, and energy level upon training and at 3-month follow-up [28]. In addition to our studies, Spijkerman et al [29] in their review and meta-analysis also reported that online mindfulness-based interventions have significant benefits on mental health outcomes, including depression (Hedges *g*=0.29), anxiety (Hedges *g*=0.22), well-being (Hedges *g*=0.23), and stress (Hedges *g*=0.51).

### Self-Compassion Training

Self-compassion training is another acceptance-based approach that has garnered empirical evidence in improving one’s well-being [30-32]. Self-compassion is defined as a caring attitude toward oneself in the face of hardship or perceived inadequacy, a recognition of suffering and failure as shared human experience, and a balanced approach to thoughts and feelings without suppression or exaggeration [33]. Self-compassionate individuals were found to bring awareness to their emotions and approach their distressing feelings with kindness and understanding, instead of avoidance and self-judgment, and they are more capable of transforming negative emotions into more positive states. Self-compassion has been demonstrated to be positively related to life satisfaction and positive affect and negatively related to negative affect, depression, and anxiety [22,34-36]. Interventions such as the 12-week Compassionate Mind Training (CMT) program [37] and 8-week Mindful Self-Compassion (MSC) program [38] have found to lead to significant increase in happiness and reduction in self-criticism, shame, sense of inferiority, stress, depression, anxiety, and global psychological distress. In addition to face-to-face training programs, researchers have used brief writing and self-help exercises to improve mental well-being. Results showed that participants reported increase in physical health, self-compassion, happiness, self-reassurance and ability to self-soothe, and decrease in depression and psychological distress [39-42].

### Exploration of Moderators

Despite evidence showing the efficacy of cognitive behavioral, mindfulness-based, and self-compassion approaches in improving mental health, little attention has been put into understanding which individual difference variables may affect the efficacy of these approaches and which can inform the choice of intervention for different individuals. Previous studies showed that people scoring high on neuroticism tend to show greater decrease in anxiety and depressive symptoms 3 months after mindfulness-based stress reduction program, whereas introverts were less likely to drop out from mindfulness-based training [43,44]. However, the underlying personality qualities and individual cognitive styles leading to differential treatment outcomes are still inconclusive. To examine who benefits from our mobile apps in this study, we hypothesized 2 moderators, specifically, discomfort with emotions and ambiguity tolerance, which might affect response to interventions.

### Discomfort With Emotions

Mindfulness and self-compassion intervention involve bringing one’s awareness to present moment emotions, either positive, negative, or neutral. Individuals with strong discomfort when experiencing emotions may have difficulties remaining in contact with such emotions. This may result in difficulty in engaging mindfulness or self-compassion practices. The study conducted by Sass et al [45] showed that reductions in distress were significantly moderated by discomfort with emotions in a brief mindfulness-based intervention. Individuals with the most discomfort with emotions showed less reduction in distress after the mindfulness-based intervention. To our knowledge, no study has examined this moderation effect in self-compassion training, but we expect to see a similar effect as compared with mindfulness-based training given that they both originated in Buddhist philosophy. On the other hand, cognitive behavioral training aims at changing the cognitive appraisal process and mood-related behaviors of the individual. While focusing on the cognitive and behavioral aspects, less emphasis was placed on experiencing and remaining in contact with one’s emotions as compared with the other 2 approaches. In this sense, a resistance toward own emotion may not affect the change mechanism as much and hence would be less likely to moderate the effect of a cognitive behavioral training.

### Ambiguity Tolerance

An important precursor to effective acceptance-based practices, including mindfulness and self-compassion, is the receptivity to new ways of being with emotional pain and suffering [46]. Van den Hurk et al [47] found that the practice of meditation is associated with higher levels of curiosity, openness, and receptivity to new experiences. This openness may be moderated by one’s level of ambiguity tolerance, which is defined as a range, from rejection to attraction, of reactions to ambiguous situations or stimuli when confronted by an array of complex, unfamiliar, or incongruent clues [48,49]. Mindfulness-based and self-compassion training rely very much on experiential learning. Thus, in these training programs, the experience may be unpredictable and variable across individuals. As mentioned earlier, it was suggested that people with low tolerance for ambiguity might benefit from a cognitive behavioral approach because of its structured context and concrete therapeutic goals, plans, and procedures [17]. Together, we hypothesized that individuals with lower levels of ambiguity tolerance may find mindfulness-based and self-compassion training more difficult to grasp than cognitive behavioral psychoeducation; thus, they are less likely to benefit from them.

### Aims and Hypotheses

Despite the fact that mindfulness, self-compassion, and cognitive behavioral approaches have garnered much evidence in their salutary effects on mental health, few studies have compared their efficacy on improving mental health in a single trial and examined how individual characteristics may affect the outcome of these intervention approaches. Moreover, most of these studies adopted the usual program format with a long program period (eg, 8 weeks to 12 weeks) and formal practices (eg, meditation that lasts for 45 min). This can be an obstacle for adults living with a packed schedule in a fast-paced city such as Hong Kong. To accommodate the local context of our target population, instead of the usual program format, our study attempted to develop and test an intervention protocol with the average engagement time being shortened to 10 to 15 min a day, for 28 days.

This study used a randomized, controlled, noninferiority trial to compare the efficacy of a 4-week mobile app–based mindfulness-based program (MBP), self-compassion program (SCP), with a cognitive behavioral psychoeducation program (CBP) in enhancing mental health among adults in Hong Kong. We hypothesized that participants in all programs will show significant and equivalent improvement in mental health at postprogram, and the changes will be maintained at 3-month follow-up. Then, we expected participants’ mindful awareness would be cultivated in both MBP and SCP but not in CBP, given the shared origin in Buddhist philosophy and the emphasis on awareness. Self-compassion was expected to be cultivated in SCP but not in the other 2 conditions. We also hypothesized that the levels of discomfort with emotions and ambiguity tolerance will post differential impact on the efficacy of the 3 respective programs. Specifically, people with lower discomfort with emotions will benefit more in CBP compared with the other 2 programs, and people with high levels of ambiguity tolerance will benefit more in both MBP and SCP compared with CBP.

## Methods

### Trial Design

This study is a 3-arm, randomized, open-label, parallel, positive-controlled trial with 3 intervention groups (MBP, SCP, and CBP). Given that the cognitive behavioral approach is a well-established, evidence-based approach for an array of mental health conditions [10], it is treated as a comparison condition that provides a more stringent evaluation of SCP and MBP as an active comparison condition and can control for demand characteristics and participant expectancies that would otherwise not be possible with a waitlist control condition. Clinical ethics approval was obtained from the principal investigator’s institution and the Hospital Authority of Hong Kong for interventions involving humans as participants. Trial registration was done through institutional registry (Trial no: ChiCTR-TRC-13003468).

### Mobile App Development

The *Living With Heart* (LWH) mobile app was developed, and it contained 3 training programs mentioned above. It runs on iOS and Android platform. A Web-browser version was also developed so that it can be accessed through various devices including mobile phones, tablets, and desktop computers. It was made available on Google Play and Apple Store, along with the website, since March 2015 after functional tests were conducted. The mobile app (and website) is fully automated and includes the following common features: (1) mood tracking function with which users can record their mood and its intensity as frequently as they wish based on either mindfulness, self-compassion, or cognitive behavioral approach that they have learned; (2) well-being tips feature with which users receive daily messages and quotes relevant to mindfulness, self-compassion, or cognitive behavioral psychoeducation, depending on to which condition the users were assigned; (3) sticker earning feature with which user can earn stickers as they progress through the sessions and they can share their accomplishments on a social networking platform such as Facebook; and (4) practice alarm feature with which users can time their practice and set timers reminding them to practice. Besides written materials, all contents have also been audio-recorded to facilitate users to listen to the content if they are unable to read the materials on the go. Screenshots of the LWH mobile app are shown in Multimedia Appendix 1.

### Interventions

In addition to the above-mentioned common features, all 3 conditions consisted of 28 daily sessions, which were divided into 4 weekly modules. The course contents were released weekly, and all 7 sessions of that particular week are available to the user on the first day of that week. Users were encouraged to read the content at their own pace with suggested home practices every week. All contents were developed by the research team members who were clinical psychologists and practitioners of cognitive behavioral, mindfulness-based, or self-compassion interventions.

#### Mindfulness-Based Program

MBP consists of 4 weekly sessions adapted from the internet-based mindfulness-based training that have been developed in the previous study [28]. Mindfulness exercises, including body scan, mindful breathing, mindful eating, mindful walking, 3-min breathing space, and thought distancing exercise [50], are audio-recorded to facilitate participants to practice mindfulness. Readings and graphics are included to explain the concept of mindfulness and to share with participants the common difficulties they may come across during mindfulness practices.

#### Self-Compassion Program

The SCP was based on the teachings of self-compassion from Neff and Germer [38]. The self-compassion exercises were adapted from the resources provided by the Center for Mindful Self-Compassion founded by Neff and Germer in 2013. Exercises included compassionate body scan, affectionate breathing, loving-kindness meditation for beginners, compassionate walking, soften-allow-soothe, self-compassion break, and self-compassion journaling. In addition to various exercises, readings and graphics were presented in each session to explain the concept of self-compassion and its relevance to mental health. Audio guides are provided to the participants to perform the self-compassion exercises.

#### Cognitive Behavioral Psychoeducation Program

In the CBP, different coping strategies and exercises to manage stress, including problem-solving skills, emotional management skills, and cognitive strategies to tackle automatic negative thoughts associated with their stress, were introduced to the participants. Relaxation skills, including abdominal breathing, progressive muscle relaxation, and imagery relaxation, were also taught with audio guides. The sessions contained information and graphics about mental health, stress, and cognitive behavioral approach to educate participants on the basic strategies to promote one’s mental health.

### Participants

This study targeted adults in the general population who fulfilled the following inclusion criteria: (1) age over 18 years, (2) read and understand Chinese, (3) own a mobile device such as a mobile phone or tablet, and (4) have consistent internet access for their mobile devices. Participants were recruited through (1) posting advertisements in free local newspapers, magazines, online advertising channels (Bing and Google Ad), and the social networking site (Facebook) and (2) sending mass emails and distributing announcements to large institutions in Hong Kong.

Participants were recruited between March 2015 and April 2016. Individuals who were interested in the study could download the mobile app through Apple Store or Google play or visit the website where informed consent was sought through the built-in consent form in the app or website. Apart from the inclusion criteria, details of the study aims, length of the program, involvement of the participants, and randomization of participants to interventions were also described. For safety, participants are reminded that the mobile app is not equivalent to a psychological treatment. They were reminded to seek professional support at any occurrence of suicidality or other medical issues. Information on help-seeking resources was provided. They were also informed that the study was conducted by the Department of Psychology at The Chinese University of Hong Kong. Individuals who agreed to participate proceeded to registration after giving informed consent by clicking the *I agree* button. From there, an activation link was sent to the participants, and they were randomly assigned to 1 of the 3 conditions.

### Randomization

Randomization took place when participants activated their user account in the email that was sent immediately to their email address after they provided informed consent on the study website. A simple randomization to 1 of the 3 conditions was performed by the computer system automatically. Participants were informed about their assigned condition after they had completed the pretraining questionnaire when they logged into the app or website.

### Measures

Participants filled in the pre-, post-, and follow-up assessments online via the website or mobile app. Trained supporters contacted the participants via a phone call and short message service text messages once after the end of the program and at 3-month follow-up to encourage the completion of postprogram and follow-up evaluations.

### Demographics

At baseline, participants were asked about their demographics and background information such as age, gender, education level, income, occupation, marital status, and religion.

### Primary Outcomes

#### Mental Well-Being

The World Health Organization 5-item Well-Being Index (WBI) [51] was used to measure mental well-being. Participants were asked to indicate how they had been feeling over the past 2 weeks on a 6-point Likert scale from 0 (*never*) to 5 (*all of the time*). In this study, its Cronbach alpha was .90 at baseline, .92 at postprogram, and .93 at 3-month follow-up.

#### Psychological Distress

The 6-item Kessler Psychological Distress Scale (K6) was used to assess psychological distress. It is a well-established screening measure on psychological distress that involves questions about a person’s emotional state. Each question is scored from 0 (*none of the time*) to 4 (*all of the time*). Its reliability and validity have been widely established across different populations [52] and in Hong Kong [53]. In this study, its Cronbach alpha was .89 at baseline, .91 at postprogram, and .90 at 3-month follow-up.

### Secondary Outcomes

#### Mindful Awareness

Five items with the highest factor loading from the Mindful Attention and Awareness Scale (MAAS) [54] were used to assess the participant’s level of mindful awareness in daily activities. Participants rate on a 6-point Likert scale ranging from 1 (*never*) to 6 (*always*). Higher scores mean having lower levels of mindful awareness. In this study, the Cronbach alpha of these items was .79 at baseline, .80 at postprogram, and .79 at 3-month follow-up.

#### Self-Compassion

To evaluate the effectiveness of the mobile app to enhance one’s self-compassion, 13 items from Self-Compassion Scale [55] were used. They were all positively framed items and were suggested to represent self-warmth in past studies [56,57]. Participants rate on a 5-point Likert scale ranging from 1 (*almost never*) to 5 (*almost always*). In this study, the Cronbach alpha of these items was .93 at baseline, .92 at postprogram, and .93 at 3-month follow-up.

### Moderators on Intervention Efficacy

#### Discomfort With Emotions

Six items with the highest factor loading from the Depressed Mood and Anxiety Subscales of the Affective Control Scale [58] that are based on the study by Melka et al [59] were used to measure discomfort with negative emotions. Cronbach alpha of these 6 items in this study at baseline, post, and follow-up were .87, .88, and .89, respectively.

#### Ambiguity Tolerance

Tolerance for ambiguity was measured by the 9-item Discomfort with Ambiguity subscale from the Need for Closure Scale [60]. Participants rated the items on a 6-point Likert scale from 1 (*strongly disagree*) to 6 (*strongly agree*). Higher scores mean having lower levels of tolerance for ambiguity. Cronbach alpha of these 9 items in this study at baseline, post, and follow-up were .75, .80, and .80, respectively.

### Program Evaluation Outcome

#### Utilization and Satisfaction

At the end of the program, participants rated on the Chinese version of the 8-item Client Satisfaction Questionnaire (CSQ) [61] for their attitudes toward and satisfaction with their assigned condition on a 4-point Likert scale. Cronbach alpha of CSQ was .87 in this study, with items 4 (“would you recommend our program to a friend”) and 8 (“would you come back to our program if you were to seek help again”) deleted due to low item-to-total correlation (item 4: *r*=−.45 and item 8: *r*=−.50). To assess the level of utilization of each participant, participants’ percentage of unlocked sessions was recorded by the backend system of the mobile app, and their retention rate in completing the post and the 3-month follow-up assessments were also recorded. Participants were also instructed to call and/or email our research assistant for clarification in case of questions, problems, or feedback during the course of the intervention.

### Analysis

All analyses were conducted using IBM SPSS 22.0. To examine and compare the efficacy between SCP, MBP, and CBP, both intention-to-treat (ITT) and per-protocol (PP) analysis were performed on the 2 primary outcome variables, that is, mental well-being and psychological distress, as well as the secondary outcome variables, that is, mindful awareness and self-compassion. For both analyses, a series of linear mixed model (LMM) analyses were conducted. Model for each outcome variable consisted of the time effect, condition effect, and the interaction effect of time by condition. First-order autoregressive covariance matrix was used. When the main effect of time or condition was significant, follow-up tests were conducted to compare the outcomes in postprogram and follow-up with the preprogram, and results were adjusted with Bonferroni correction.

In handling longitudinal missing data, Newman [62] has found in a series of simulation that maximum likelihood and multiple imputation approaches yielded better SE estimates than other approaches. In addition, it has been suggested that restricted maximum likelihood (and full information maximum likelihood) is superior to multiple imputation approach in estimating SE when handling missing data with second-level dependencies [63]. In this study, we handled missing data using the restricted maximum likelihood approach to better account for the missing data that involved second-level dependencies.

Effect sizes (ie, Cohen *d*) of each intervention were calculated by subtracting the postscore or follow-up score of each outcome measure from the respective prescore and then dividing the difference by the pooled SD [64]. Moderation was examined by the same LMM procedure described above, and the model consisted of the main effect of time, condition, and the moderator, the 2-way interaction effects (ie, time x condition, time x moderator, condition x moderator), and the 3-way interaction effect (ie, time x condition x moderator). A significant 3-way interaction effect (time x condition x moderator) indicates moderation effect.

## Results

### Recruitment and Participant Characteristics

**Figure 1:**
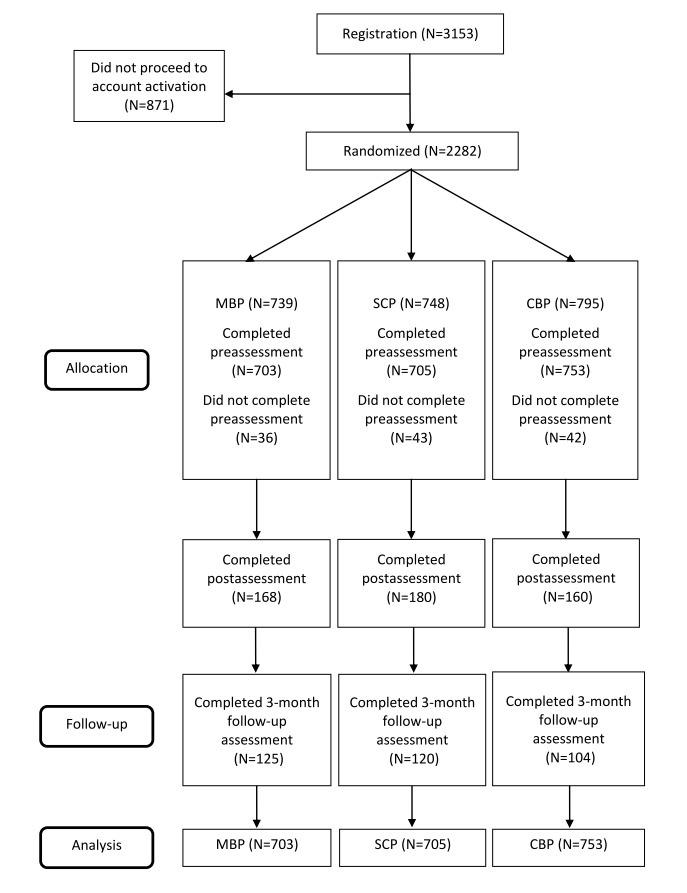
CONSORT flowchart of participants in our study. MBP: mindfulness-based program; SCP: self-compassion program; CBP: cognitive behavioral psychoeducation program.

A total of 3153 registrants had downloaded the mobile app and registered an account. A total of 27.62% (871/3153) chose not to activate their accounts, whereas 2282 registrants proceeded with account activation followed by randomization. Among those who proceeded to registration, 739 were randomized to the MBP, 748 to the SCP, and 795 to the CBP. Furthermore, 95.1% (703/739) randomized participants in the MBP, 94.3% (705/748) in the SCP, and 94.7% (753/795) in the CBP completed the prequestionnaire and began the program (see Figure 1 for study flowchart).

Demographics and baseline psychological attributes of the participants are shown in Tables 1 and 2. Overall, they had a mean age of 33.64 (SD 12.08), with the majority being female (72.88%, 1575/2161), and 79.59% (1720/2161) received or were receiving tertiary education (undergraduate or above).

### Utilization Analysis

The mean completion rate of the 28 sessions (4 modules) of all participants (including completers and noncompleters) was 31.95% (SD 34.94), approximately 9 out of 28 days. The mean completion rate for MBP was 29.48% (SD 34.23), 32.15% (SD 34.72) for SCP, and 34.08% (SD 34.13) for CBP. The 3 conditions differ significantly on the overall progress, *F*_2_=3.272, *P*=.04*.* Follow-up test showed that the progress was significantly greater in CBP than in MBP (*P*=.03).

**Table 1:** Baseline characteristics across conditions.

Characteristics		MBP^a^ (N=703)	SCP^b^ (N=705)	CBP^c^ (N=753)
**Age in years**				
	Mean (SD)	33.80 (12.40)	33.59 (11.91)	33.54 (11.95)
	Range	18-83	18-69	18-68
**Gender, n (%)**				
	Male	203 (28.9)	199 (28.2)	184 (24.4)
	Female	500 (71.1)	506 (71.8)	569 (75.6)
**Education, n (%)**				
	Primary or below	10 (1.4)	8 (1.1)	7 (0.9)
	Secondary	143 (20.4)	125 (17.7)	148 (19.7)
	Bachelor/diploma	385 (54.7)	379 (53.8)	405 (53.8)
	Master or above	165 (23.4)	193 (27.4)	193 (25.6)
**Employment, n (%)**				
	Student	184 (26.6)	179 (25.9 )	201 (27.3)
	Full-time	377 (54.5)	394 (57.0)	394 (53.5)
	Part-time	42 (6.1)	29 (4.2)	27 (3.7)
	Unemployed	24 (3.5)	33 (4.8)	55 (9.3)
	Others	76 (9.3)	70 (8.1)	76 (6.2)
**Religion, n (%)**				
	No religion	422 (60)	410 (58.5)	424 (56.8)
	Christians	169 (24)	183 (26.1)	200 (26.8)
	Catholics	40 (5.7)	43 (6.1)	51 (6.8)
	Buddhists	64 (9.1)	57 (8.1)	63 (8.4)
	Others	8 (1.1)	8 (1.1)	8 (1.1)
**Previous mindfulness experience, n (%)**				
	Yes	611 (86.9)	599 (85)	629 (83.5)
	No	92 (13.1)	106 (15)	124 (16.5)
**Previous CBT^d^ experience, n (%)**				
	Yes	638 (90.8)	626 (88.8)	661 (87.8)
	No	65 (0.1)	79 (11.2)	92 (12.2)

^a^MBP: mindfulness-based program.

^b^SCP: self-compassion program.

^c^CBP: cognitive behavioral psychoeducation program.

^d^CBT: cognitive behavioral therapy.

**Table 2:** Baseline characteristics across conditions.

Outcome measures		MBP^a^ (N=703), mean (SD)	SCP^b^ (N=705), mean (SD)	CBP^c^ (N=753), mean (SD)
**Primary outcomes**				
	Mental well-being	3.00 (0.99)	2.99 (1.02)	2.99 (1.00)
	Psychological distress	2.43 (0.88)	2.44 (0.83)	2.48 (0.86)
**Secondary outcomes**				
	Mindful awareness	2.50 (0.91)	2.51 (0.88)	2.59 (0.93)
	Self-compassion	2.82 (0.78)	2.79 (0.80)	2.80 (0.80)
**Potential moderators**				
	Discomfort with emotions	4.29 (1.14)	4.32 (1.14)	4.35 (1.14)
	Ambiguity tolerance	4.36 (0.67)	4.38 (0.71)	4.36 (0.67)

^a^MBP: mindfulness-based program.

^b^SCP: self-compassion program.

^c^CBP: cognitive behavioral psychoeducation program.

**Figure 2:**
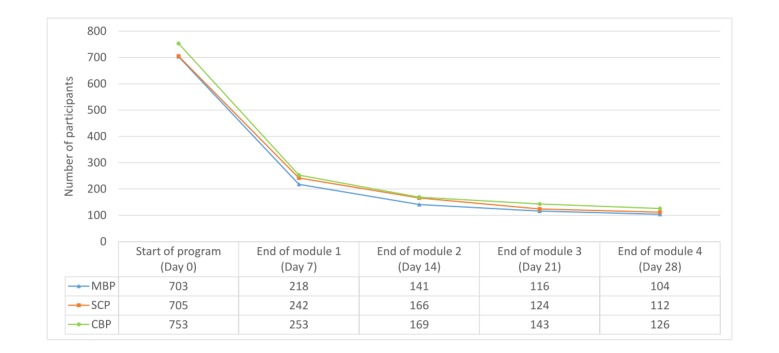
Number of participants using the programs each week. MBP: mindfulness-based program; SCP: self-compassion program ; CBP: cognitive behavioral psychoeducation program.

Figure 2 shows the number of participants who stayed in the program after each module. Numbers indicated that the majority of attrition was noted in the first week. Specifically, 69.0% (485/703) of participants in the MBP, 65.7% (463/705) in the SCP, and 66.4% (500/753) in the CBP stopped using the app after 7 days.

### User Experience

Participants of the 3 conditions (MBP, SCP, and CBP) reported similar overall usage satisfaction as measured by the CSQ after removing item 4 and 8, *F*_2_=2.319, *P*=.10. Of the 508 users who responded to the CSQ, 79.9% (406/508) found the course contents good or excellent. In addition, 90.2% (458/508) stated that they generally or definitely got the service (learning experience) that they wanted. More than half of the participants, 56.1% (285/508), thought the program met most or almost all of their needs. Moreover, 88.0% (447/508) of users were mostly or very satisfied with the amount of help received in the program and 77.8% (395/508) found it somewhat helpful in dealing with their problems more effectively. Furthermore, 87.4% (444/508) of our participants were mostly or very satisfied with the mobile app in general.

### Attrition Analysis

To investigate the potential causes of attrition, we compared the baseline attributes between participants who dropped-out (N=1653) with those who remained (N=508) at postprogram. Participants who stayed in the program (mean 34.75 SD 12.76) were significantly older than those who left (mean 33.3 SD 11.84), *t*_794.29_=2.27, *P*=.02. They also differed in terms of education level, χ^2^_6_=14.23, *P*=.03, with more people obtaining postgraduate education in the dropout group. No significant difference was found in all outcome measures and potential moderators at baseline.

### Intent-to-Treat Analysis Findings

#### Mental Well-Being

Results from LMM analyses found that the mobile apps significantly enhanced participants’ well-being overtime, *F*_2_=51.36, *P*<.001. Scores on WBI significantly increased from baseline to postprogram (mean difference=0.31, 95% CI 0.22-0.40, *P*<.001) and from baseline to 3-month follow-up (mean difference=0.35, 95 CI 0.24-0.46, *P*<.001). There is no significant main effect of condition (*P*=.43). The nonsignificant time x condition interaction effect (*P*=.67) indicated that the improvements over time were identical across the 3 conditions.

#### Psychological Distress

Psychological distress as measured by the K6 was found to be significantly reduced in all 3 conditions, *F*_2_=44.60, *P*<.001. Mean score of K6 significantly decreased from baseline to postprogram (mean difference=−0.26, 95% CI −0.33 to −0.19, *P*<.001), and this decrease was maintained at 3-month follow-up (mean difference=−0.22, 95% CI −0.31 to −0.13, *P*<.001) in all 3 conditions. No significant main effect of condition (*P*=.72) and interaction term (*P*=.52) was noted.

#### Mindful Awareness

The mobile apps significantly enhanced participants’ mindful awareness over time, *F*_2_=4.94, *P*<.01. Mean scores of MAAS significantly decreased from baseline to postprogram in all 3 conditions (mean difference=−0.11, 95% CI −0.19 to −0.03, *P*<.01). However, the change from baseline to follow-up was not significant (mean difference=−0.04, 95% CI −0.13 to .06, *P*>.99). The main effect of condition was not significant (*P*=.09). There is no significant time x condition interaction effect (*P*=.59) as well.

### Self-Compassion

A significant time x condition interaction effect was found, *F*_4_=2.72, *P*<.05. This indicated a different change pattern across the 3 conditions. This interaction was followed up by post-hoc comparisons. We found that both SCP (mean difference=0.25, 95% CI 0.14-0.36, *P*<.001) and CBP (mean difference=0.21, 95% CI 0.09-0.32, *P*<.001) were able to enhance self-compassion at postprogram. MBP did not significantly improve self-compassion at postprogram (mean difference=0.06, 95% CI −0.06 to 0.17, *P*=.70). None of the conditions significantly improved self-compassion from baseline to 3 months after adjustment. It is noteworthy that the change in self-compassion from baseline to 3 months in MBP was approaching significance (*P*=.055).

### Per-Protocol Analysis Findings

The PP population was defined as all participants who have completed all 28 days (100%) of the program. There were a total of 342 participants in this population, 104 in the MBP, 112 in the SCP, and 126 in the CBP group. Results of the PP analysis on the 2 primary outcomes were similar to the full sample analysis. Slight differences were found in the results regarding the 2 secondary outcomes.

#### Mental Well-Being

The mobile apps significantly enhanced participants’ mental well-being over time, *F*_2_=31.47, *P*<.001. Scores on WBI significantly increased from baseline to postprogram (mean difference=0.37, 95% CI 0.24-0.49, *P*<.001) and from baseline to 3-month follow-up (mean difference=0.43, 95% CI 0.27-0.59, *P*<.001). There is no significant main effect of condition (*P*=.98). The nonsignificant time x group interactions (*P*=.68) indicated that the improvements over time were identical across the 3 conditions.

#### Psychological Distress

Psychological distress as measured by the K6 was found to be significantly reduced over time, *F*_2_=27.57, *P*<.001. Mean scores of K6 significantly decreased from baseline to postprogram (mean difference=−0.30, 95% CI −0.40 to −0.19, *P*<.001) and from baseline to 3-month follow-up (mean difference=−0.27, 95% CI −0.40 to −0.15, *P*<.001). The main effect of condition was not significant (*P*=.12). There is no significant time x condition interaction effect (*P*=.63).

#### Mindful Awareness

Results in this PP analysis showed a nonsignificant main effect of time, *F*_2_=1.92, *P*=.15. However, the condition effect was significant, *F*_2_=3.53, *P*<.05. Follow-up tests showed that the mean MAAS score in SCP was significantly differed from that of CBP (mean difference=0.27, 95% CI 0.03-0.52, *P*<.05). Specifically, MAAS scores in CBP (mean 2.613, SE 0.07, 95% CI 2.47-2.75) were higher than those in SCP (mean 2.34, SE 0.07, 95% CI 2.20-2.49), but did not significantly differ from that of MBP (mean 2.48, SE 0.08, 95% CI 2.33-2.63). The time x condition interaction effect was not significant (*P*=.95).

#### Self-Compassion

A significant time x condition interaction effect was found, *F*_4_=2.52, *P*<.05. In the follow-up test, we found that both SCP (mean difference=0.34, 95% CI 0.17-0.50, *P*<.001) and CBP (mean difference=0.20, 95% CI 0.04-0.36, *P*<.05) were able to enhance self-compassion at postprogram, but not MBP (*P*>.99). None of the conditions significantly improved self-compassion from baseline to 3 months after adjustment.

Details of the ITT and PP analyses are shown in Tables 3 and 4.

### Findings on Potential Moderation Effects

Results revealed that the proposed moderators did not moderate the effect of intervention efficacy in terms of WBI. The moderation of discomfort with emotions (*F*_4_=0.60, *P*=.66) and ambiguity tolerance (*F*_4_=1.40, *P*=.23) were not significant. Similarly, for distress reduction (K6), no significant interaction was noted. The moderation of discomfort with emotions (*F*_4_=0.55, *P*=.70) and ambiguity tolerance (*F*_4_=0.62, *P*=.65) were not significant.

**Table 3:** Means, SD, and SE of primary outcomes across conditions.

Outcome measures		MBP^a^, mean (SD^d^; SE)			SCP^b^, mean (SD^d^; SE)			CBP^c^, mean (SD^d^; SE)		
		Pre	Post	FU^e^	Pre	Post	FU	Pre	Post	FU
N (ITT^f^)		703	168	125	705	180	120	753	160	104
N (PP^g^)		104	88	61	112	95	61	126	98	58
**Mental well-being**										
	WBI^h^ (ITT)	3.00 (1.06; 0.04)	3.32 (0.91; 0.07)	3.42 (0.89; 0.08)	2.99 (1.06; 0.04)	3.25 (0.80; 0.06)	3.26 (0.88; 0.08)	2.99 (1.10; 0.04)	3.34 (0.89; 0.07)	3.45 (0.82; 0.08)
	WBI (PP)	3.02 (1.02; 0.10)	3.32 (1.03; 0.11)	3.54 (0.94; 0.12)	3.01 (1.06; 0.10)	3.42 (0.97; 0.10)	3.45 (0.94; 0.12)	3.05 (1.01; 0.09)	3.41 (0.99; 0.10)	3.44 (0.91; 0.12)
**Psychological distress**										
	K6^i^ (ITT)	2.43 (0.80; 0.03)	2.20 (0.65; 0.05)	2.18 (0.67; 0.06)	2.44 (0.80; 0.03)	2.15 (0.67; 0.05)	2.23 (0.77; 0.07)	2.48 (0.82; 0.03)	2.23 (0.76; 0.06)	2.23 (0.71; 0.07)
	K6 (PP)	2.51 (0.82; 0.08)	2.15 (0.84; 0.09)	2.15 (0.78; 0.10)	2.33 (0.85; 0.08)	2.07 (0.78; 0.08)	2.15 (0.78; 0.10)	2.57 (0.90; 0.08)	2.30 (0.79; 0.08)	2.29 (0.69; 0.09)
**Mindful awareness**										
	MAAS^j^ (ITT)	2.50 (0.80; 0.03)	2.43 (0.78; 0.06)	2.42 (0.78; 0.07)	2.51 (0.80; 0.03)	2.35 (0.80; 0.06)	2.49 (0.77; 0.07)	2.59 (0.82; 0.03)	2.50 (0.76; 0.06)	2.59 (0.71; 0.07)
	MAAS (PP)	2.56 (0.92; 0.09)	2.44 (0.84; 0.09)	2.45 (0.78; 0.10)	2.40 (0.85; 0.08)	2.29 (0.88; 0.09)	2.34 (0.78; 0.10)	2.63 (0.90; 0.08)	2.57 (0.89; 0.09)	2.64 (0.76; 0.10)
**Self-compassion**										
	SCS (ITT)	2.82 (0.80; 0.03)	2.88 (0.65; 0.05)	2.96 (0.67; 0.06)	2.79 (0.80; 0.03)	3.03 (0.67; 0.05)	2.91 (0.66; 0.06)	2.80 (0.82; 0.03)	3.00 (0.63; 0.05)	2.94 (0.61; 0.06)
	SCS (PP)	2.78 (0.82; 0.08)	2.82 (0.75; 0.08)	2.91 (0.70; 0.09)	2.83 (0.80; 0.08)	3.17 (0.78; 0.08)	3.00 (0.62; 0.08)	2.85 (0.79; 0.07)	3.05 (0.79; 0.08)	3.00 (0.69; 0.09)

^a^MBP: mindfulness-based program.

^b^SCP: self-compassion program.

^c^CBP: cognitive behavioral psychoeducation program.

^d^SD was computed from SE multiplied by the square root of sample size.

^e^FU: follow-up.

^f^ITT: intent-to-treat analysis.

^g^PP: per-protocol analysis.

^h^WBI: well-being index.

^i^K6: Kessler Psychological Distress Scale.

^j^MAAS: Mindful Attention and Awareness Scale.

In light of the Buddhist origin of our interventions, we also tested whether there is any differential effect of participants’ religion on mental well-being and psychological distress. It was found that the changes in mental well-being and psychological distress did not differ significantly by religion, as revealed by the nonsignificant 3-way interaction effects in the LMM analyses with religion as the covariate (WBI: *P*=.55; K6: *P*=.79).

In addition, as there was a large difference in the sample size of males and females, we therefore examined if there are any potential moderating effect of participants’ gender. It was found that the changes in well-being and psychological distress did not differ significantly across the 2 genders, as revealed by the nonsignificant 3-way interaction effects in the LMM analyses with gender as the covariate (WBI: *P*=.57; K6: *P*=.54).

**Table 4:** Summary of time effects and effect sizes across conditions.

Outcome measures		MBP^a^, Cohen *d*^d^		SCP^b^, Cohen *d*^d^		CBP^c^, Cohen *d*^d^		Overall time effect			
		Post^e^	FU^e,f^	Post^e^	FU^e^	Post^e^	FU^e^	Post vs pre, mean difference (95% CI)	*P* value	FU vs pre, mean difference (95% CI)	*P* value
**Mental well-being**											
	WBI^g^ (ITT^h^)	0.32	0.42	0.27	0.28	0.35	0.36	0.31 (0.22 to 0.40)	<.001	0.35 (0.24 to 0.46)	<.001
	WBI (PP^i^)	0.31	0.51	0.40	0.40	0.36	0.38	0.37 (0.24 to 0.49)	<.001	0.43 (0.27 to 0.59)	<.001
**Psychological distress**											
	K6^j^ (ITT)	−0.28	−0.30	−0.35	−0.19	−0.31	−0.30	−0.26 (−0.33 to −0.19)	<.001	−0.22 (−0.31 to −0.13)	<.001
	K6 (PP)	−0.44	−0.44	−0.31	−0.22	−0.32	−0.34	−0.30 (−0.40 to −0.19)	<.001	−0.27 (−0.40 to −0.15)	<.001
**Mindful awareness**											
	MAAS^k^ (ITT)	−0.08	−0.10	−0.19	−0.03	−0.10	0	−0.11 (−0.19 to −0.03)	<.01	−0.04 (−0.13 to 0.06)	>.99
	MAAS (PP)	−0.13	−0.11	−0.13	−0.08	−0.08	0.01	−0.10 (−0.22 to 0.02)	.16	−0.05 (−0.19 to 0.08)	>.99
**Self-compassion**											
	SCS^l^ (ITT)	0.07	0.17	0.32	0.16	0.27	0.18	0.17 (0.11 to 0.23)	<.001	0.13 (0.05 to 0.21)	<.001
	SCS (PP)	0.06	0.16	0.43	0.22	0.25	0.20	0.19 (0.10 to 0.29)	<.001	0.15 (0.03 to 0.27)	<.05

^a^MBP: mindfulness-based program.

^b^SCP: self-compassion program.

^c^CBP: cognitive behavioral psychoeducation program.

^d^Cohen *d* was computed from postprogram/3-month follow-up score minus preprogram score divided by the pooled SD.

^e^Versus pre.

^f^FU: follow-up.

^g^WBI: well-being index.

^h^ITT: intent-to-treat analysis.

^i^PP: per-protocol analysis.

^j^K6: Kessler Psychological Distress Scale.

^k^MAAS: Mindful Attention and Awareness Scale.

^l^SCS: Self-Compassion Scale.

## Discussion

### Principal Findings—Efficacy and Application

Despite the fact that numerous mental health–related mobile apps are in the market, many of them have not been empirically tested. Even when tested, these studies often employed case studies and prepost design. This study was one of the few studies that used a rigorous RCT design in comparing the efficacy of mobile mindfulness-based training and self-compassion training with an evidence-based cognitive behavioral psychoeducation app. We also examined potential moderators that may affect training outcomes of the 3 conditions. Results demonstrated that the use of the 4-week MBP, SCP, or CBP led to significant improvement in mental well-being and reduced psychological distress in our participants who completed the online assessments, and these improvements were sustained at 3-month follow-up. The effect sizes obtained in this study were small to moderate on the primary outcomes (*d*=−0.19 to 0.51). They were comparable with other online mindfulness-based interventions in improving mental health [29]. Nonetheless, the effect sizes in our online self-compassion and mindfulness-based programs (as well as in CBP) were comparable with other unguided internet-based CBT trials, for instance, Berger et al [65], supporting these approaches to be noninferior to other unguided internet-based CBTs.

With 1 in 7 adults having a common mental disorder and only 1 in 4 of them seeking formal mental health services in Hong Kong [3], a population-based approach is likely to have the greatest impact in reducing mental health burden in the community [66]. In comparison with face-to-face interventions, mobile app interventions are easily accessible and have the potential to meet the need for mental health promotion and universal prevention in the community settings. This study showed that app-based mental health training programs are viable strategies that can be easily incorporated into existing service provision portfolios in promoting mental health in the general population.

This study opted for a noninferiority design that employed a cognitive behavioral psychoeducation control program instead of a waitlist or placebo control. Previous studies have demonstrated the efficacy of internet-based mindfulness-based training on well-being compared with waitlist control [27] and with CBP training [28]. Self-compassion training is also found to promote well-being compared with waitlist control in face-to-face setting [39,67]. We posit this approach as a logical scientific extension of the existing literature by showing the efficacy of a mobile app–based MBP, SCP, and CBP in the promotion of mental health. Besides, it is practically difficult to put participants on a waitlist control and withhold program content from them. As the mobile app was published on the app market (Apple store and Google Play) during the study period, everyone in the public could access and download the app freely.

Results showed that mindfulness-based training and self-compassion training was as efficacious as the CBP active comparison condition. Such comparable findings were encouraging. Cognitive behavioral training has been widely studied in the literature and has demonstrated its efficacy and effectiveness in managing psychological distress [10] and enhancing well-being in the general population [14]. The fact that mindfulness-based training and self-compassion training showed comparable improvement in mental health outcomes provided the public alternative evidence-based options to promote their mental well-being.

### Cultivation of Mindful Awareness and Self-Compassion

Our secondary hypotheses on mindful awareness and self-compassion were only partially supported. In the ITT analysis, both MBP and SCP enhanced participants’ mindful awareness at postprogram as expected. The benefit in terms of mindful awareness enhancement in MBP participants was intuitive. As for SCP, the improvement was also in line with the literature. Neff [21] asserts that mindfulness is 1 of the 3 facets in forming self-compassion. It involves holding one’s painful thoughts and feelings in balanced awareness rather than over-identifying with them. Although not explicitly stated, the SCP modules inevitably involved mindfulness concepts. The meditation exercises in the SCP, for instance, self-compassion breathing exercise and loving-kindness meditation, also required participants to focus on the moment-by-moment experiences and therefore enhanced participants’ awareness.

Specifically, we did not find that mindfulness condition showed significantly greater improvement in the levels of mindfulness over time as compared with the improvement in SCP and CBP conditions. Similarly, Turner et al [68] also found that mindfulness-based stress reduction program did not show significantly greater improvement in mindfulness compared with the CBT condition among people with chronic lower back pain. The authors postulated that although CBT reduced catastrophizing through cognitive restructuring techniques, mindfulness might improve indirectly as a result. Similarly, in this study, some specific mediators were not measured in the study, including acceptance to painful experience in the SCP condition or reduced catastrophizing of experience in the CBP condition, which may affect levels of mindfulness. More research is needed to examine the mechanisms behind the change.

Similarly, the enhanced self-compassion in CBP participants was also unexpected. However, self-compassion may be related to unhealthy perfectionism. According to CBT models, unhealthy perfectionism was maintained by negatively biased thinking patterns such as self-critical thinking and self-imposed “should” and “musts” statements. These, in turn, contributed to an elevated self-criticism in oneself [69]. There was evidence that unguided self-help using CBT approach can reduce perfectionism [70]. A recent study [71] also found that the use of a CBT self-help booklet significantly improved participants’ self-compassion, although to a lesser degree compared with mindfulness-based cognitive therapy. Our findings added to this area of literature.

In addition, the differential change profiles of self-compassion across the 3 groups also caught our attention. When taking a closer look into the trends, self-compassion greatly improved and then gradually went down in SCP and CBP, whereas in MBP, self-compassion increased to a lesser extent after the program but gradually went up at 3-month, and this change approached significance (*P*=.05). Although mindfulness is the prerequisite of forming self-compassion as suggested by Neff [21], MBP participants who are trained in mindfulness may catalyze the cultivation of self-compassion in the long term. Future studies are warranted to test this speculation.

Another observation was that both the changes in mindful awareness and self-compassion did not sustain through the 3-month period. This is possibly due to an absence of practice reminder after the 28-day program. It is well accepted that mindfulness and self-compassion meditation requires persistent and long-term practice for it to be effective. We acknowledged that, however, the improvements in mental health were maintained, indicating that there may be other factors mediating the changes in mental health in our participants. These underlying factors need to be further explored in future studies.

### Exploration of Moderators

Contrary to our hypotheses, the proposed moderators (discomfort with emotions and tolerance for ambiguity) did not appear to moderate the training effects. Reasons may be that all the measures used to tap onto the constructs are abridged versions to keep the brevity of the online questionnaire. Although all the measures had satisfactory internal consistency, their validity in measuring the intended constructs needs to be further investigated and confirmed. Given the findings of this study, we have no empirical evidence pointing to the suitability of different training programs for different types of populations. The general population seems to be equally responsive to mindfulness-based, self-compassion, and cognitive behavioral psychoeducation training. However, Teper et al [72] proposed that mindfulness-based training facilitated adaptive emotion regulation by fostering interoceptive awareness. Therefore, it seems reasonable to postulate that contemplative training involving mindfulness and self-compassion may deem more intrinsically rewarding for individuals who are interoceptive and introspective to begin with. Future studies should continue examining other possible moderating effects (eg, interoception and introspection) to better match users to programs that are compatible with their individual differences and preferences.

### Limitations and Future Directions

Several limitations need to be considered when interpreting the results. First, the attrition rate of this study was high (76.5% at postprogram and 83.9% at 3-month follow-up). It must be recognized that high attrition has been a common concern shared by many internet-based intervention studies. For example, Mitchell et al [73] reported an attrition rate of 83% in their well-being promotion trial. In a systematic review of internet-based interventions for anxiety and depression, the completion rates ranged from 43% to 99% [74]. The use of an unguided self-help approach may have contributed to the low retention rate as well. Previous studies comparing guided versus unguided self-help approaches have reported higher adherence rate in guided interventions [75,76]. Although unguided self-help can reduce the cost and labor in providing coaching or guidance, it may compromise adherence and overall efficacy of the training program [77]. The unguided nature of the interventions may also explain these small effect sizes as other studies on unguided self-help also reported small to medium effect sizes [78].

Furthermore, the younger participants were more prone to withdraw from the study. This might affect the generalizability of our findings. The characteristics of dropouts are, however, closely consistent with Mispel et al [79] in their recent investigation of user characteristics in relation to attrition. They also found that male users and younger adults were more likely to quit an online intervention.

We noted the importance of users’ experience in the initiation phase as it was observed that most participants who dropped out ceased using the apps within the first 7 days. This provides indications for future mHealth interventions, especially when the apps will be freely accessible in the app market where people can download and try using them without monetary cost. Researchers should pay attention to users’ experience alongside the course contents when designing the apps. Future studies can consider gamification [80,81] or personalization of feedback [82] to enhance its appeal to the participants and increase the personal relevance of the training to each participant as well as the inclusion of online coaches or guidance to support the users during the course of the training.

Another limitation was that the inferior design of this study precluded us from ruling out the possibility of a placebo effect in explaining the improvement in mental health among our participants. To rule out the placebo effect, future studies should consider building a placebo control condition in the app such as reading an electronic book not related to psychology, but this may increase the cost of developing an additional placebo condition for the study.

Participants in this study by nature skewed toward people who are proficient in using computer or mobile devices. There is a possibility that these people might be more educated. The latest government statistics [83] revealed that nearly half of the Hong Kong citizens (49.7%) received up to secondary education, whereas in our sample, more than half of our participants were receiving tertiary education. These participants may have higher mental health literacy and be more open to participate in mental health programs. This limits the generalizability of our findings to all segments of the population (eg, less educated individuals, low income), even though Hong Kong has the highest penetration rate of mobile devices in the world [4]. Future studies should focus on how mobile app–based interventions can cater to different segments of the populations through various adaptations.

### Conclusions

In total, the LWH mobile app was tested in this study and mindfulness-based, self-compassion, and cognitive behavioral training programs were found to be efficacious in promoting mental health and reducing psychological distress among adults in Hong Kong who used the app. Given the mental health burden in our communities, this study showed that mobile-based interventions can be an option for mass dissemination in improving public mental health.

## Appendix

Multimedia Appendix 1 Screenshots of the LWH application.

## Abbreviations

CBP: cognitive behavioral psychoeducation program
CBT: cognitive behavioral therapy
CSQ: Client Satisfaction Questionnaire
ITT: intention-to-treat
K6: Kessler Psychological Distress Scale
LMM: linear mixed model
LWH: “Living With Heart” mobile app
MAAS: Mindful Attention and Awareness Scale
MBP: mindfulness-based program
PP: per-protocol
SCP: self-compassion program
WBI: well-being index
