# Supplementary material for: Efficacy and Moderation of Mobile App–Based Programs for Mindfulness-Based Training, Self-Compassion Training, and Cognitive Behavioral Psychoeducation on Mental Health: Randomized Controlled Noninferiority Trial
Source: JMIR Ment Health. 2018 Oct 11;5(4):e60. doi: 10.2196/mental.8597 (PMC6231823; doi:10.2196/mental.8597)
Supplement: Multimedia Appendix 1 [file mental_v5i4e60_app1.pdf]

Multimedia Appendix 2: Screenshots of the “Living With Heart” Application

Part 1: Screenshots from sign-in page to course enrolment

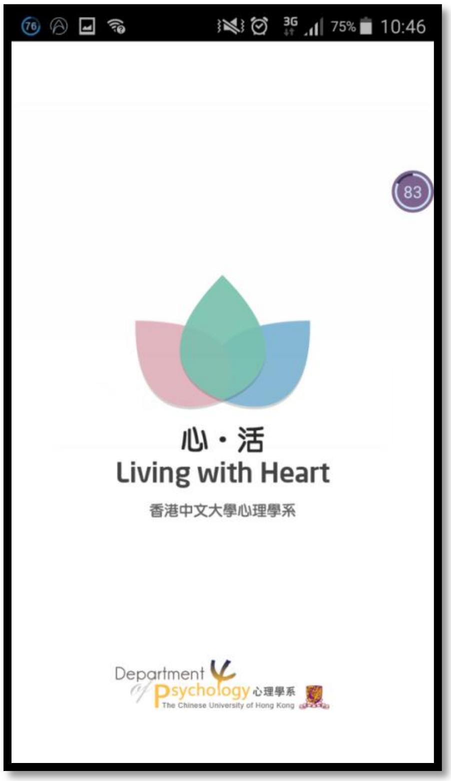

Description: Landing page

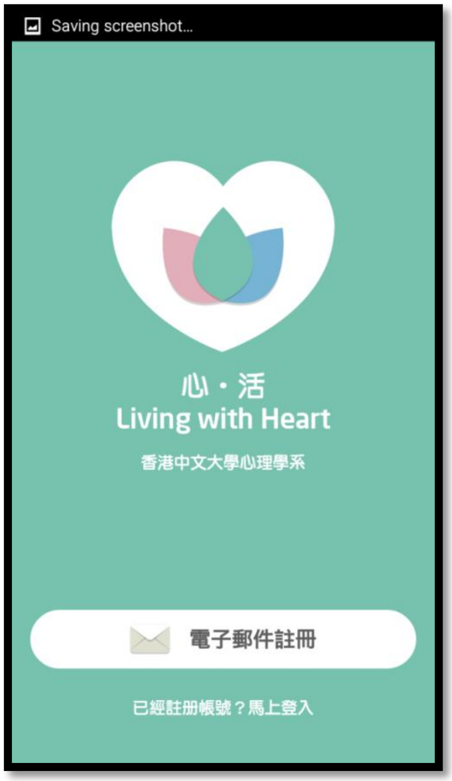

Description: Registration by email address

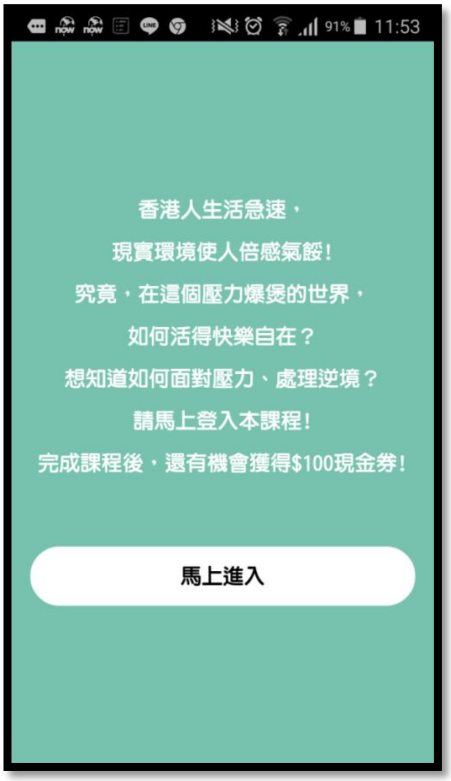

Description: Forewords and a button written: “Start now”

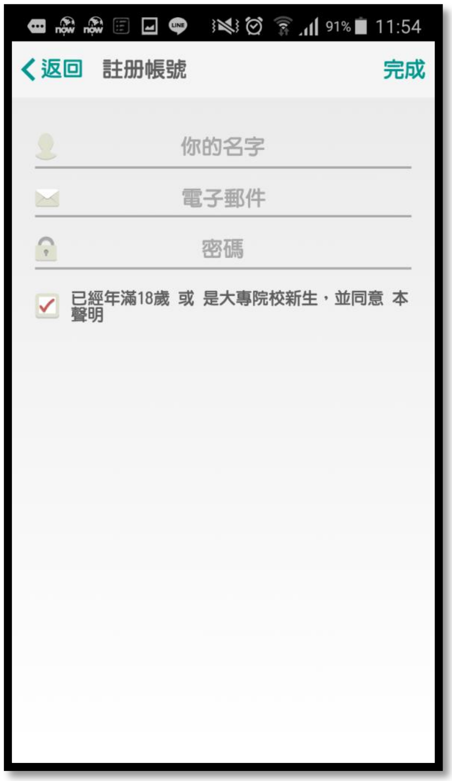

Description: Registration, asking for user ID, email address and create password

Multimedia Appendix 2: Screenshots of the “Living With Heart” Application

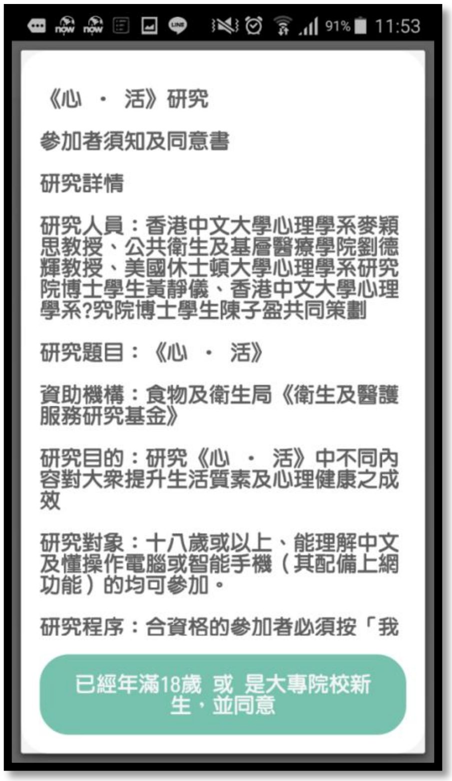

Description: Informed Consent. A button written: “I’m over 18 or a college student and I agree to join”

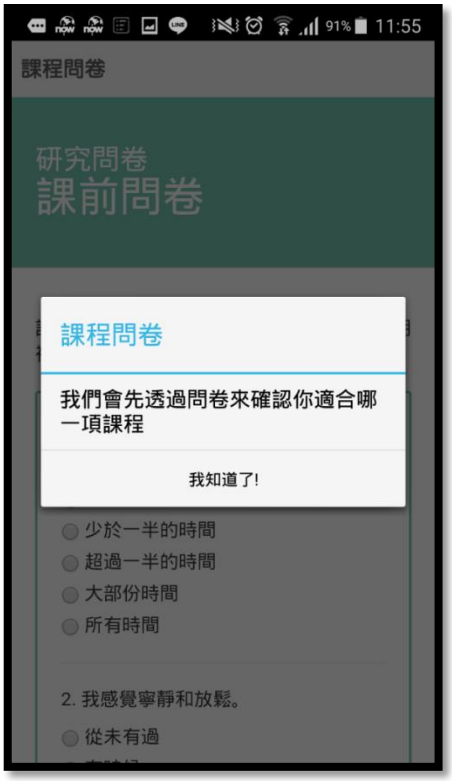

Description: Pre-programme questionnaires

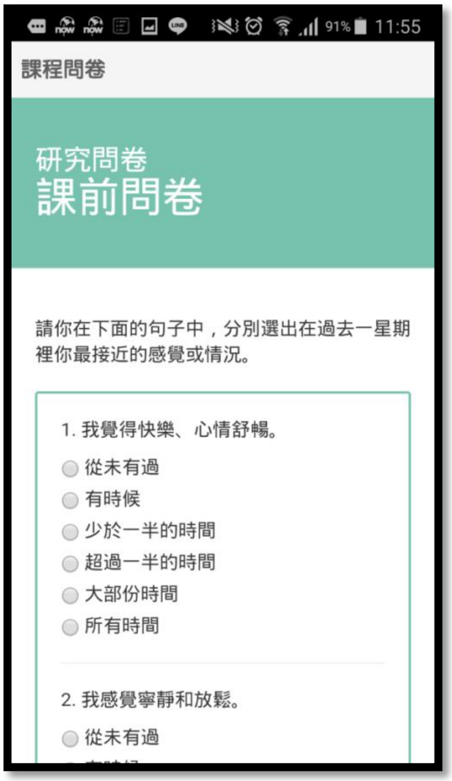

Description: Sample of questionnaires

Multimedia Appendix 2: Screenshots of the “Living With Heart” Application

Part 2. Screenshots of course content

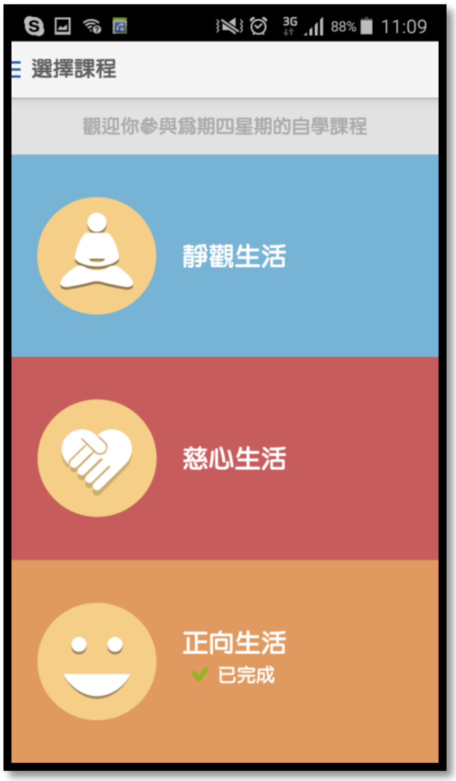

Description: The 3 built-in courses in the LWH application: MBT (Blue), SCP (Red) and CBP (Orange).

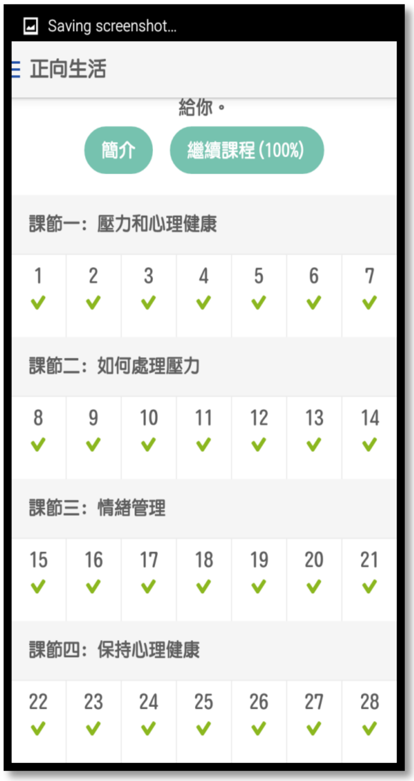

Description: There are a total of 4 modules (28 sessions) in each course. A green tick represents a completed session.

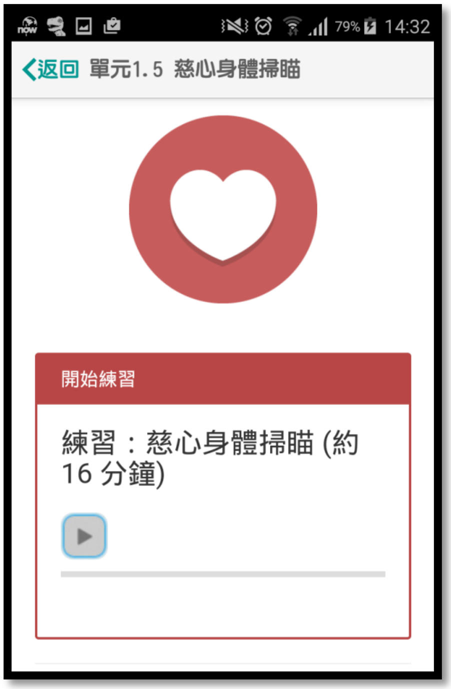

Description: Audio exercises (e.g. compassionate body scan)

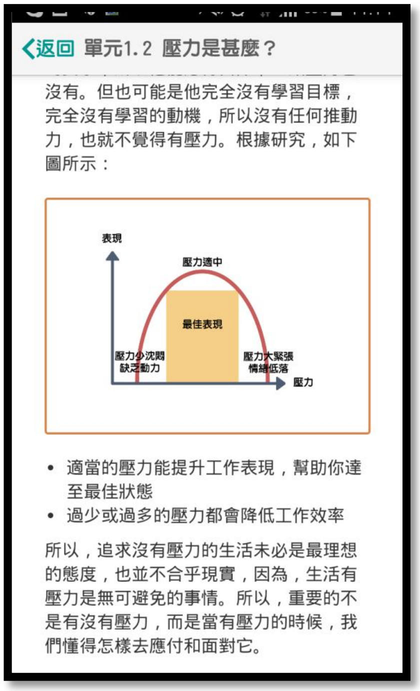

Description: Sample contents in CBP

Multimedia Appendix 2: Screenshots of the “Living With Heart” Application

Part 3. Sample of features and tools

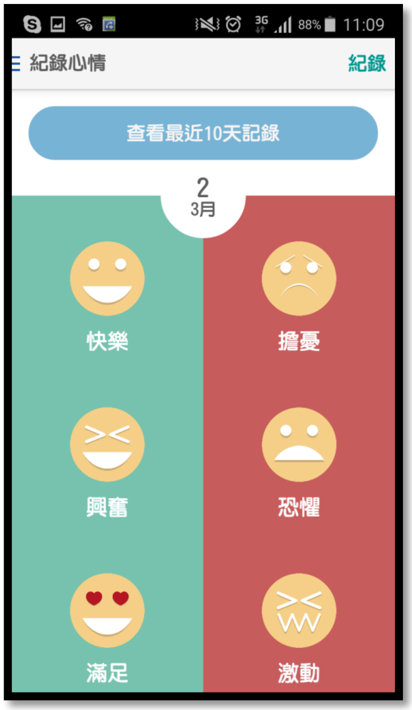

Description: Mood tracking function

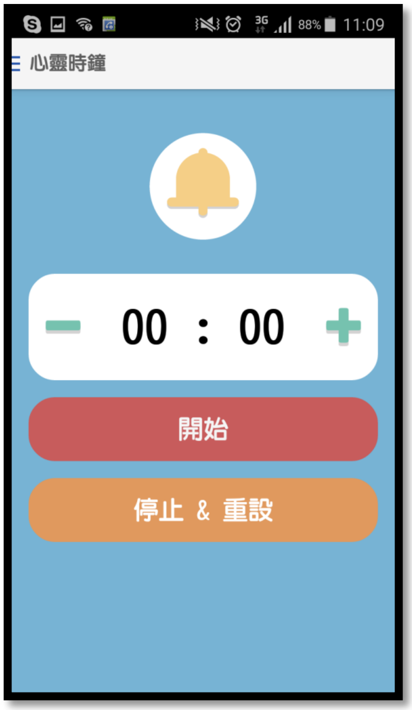

Description: Practice alarm

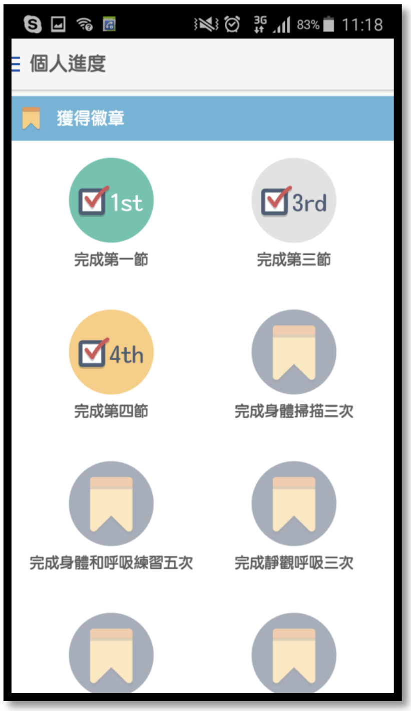

Description: sticker earning feature

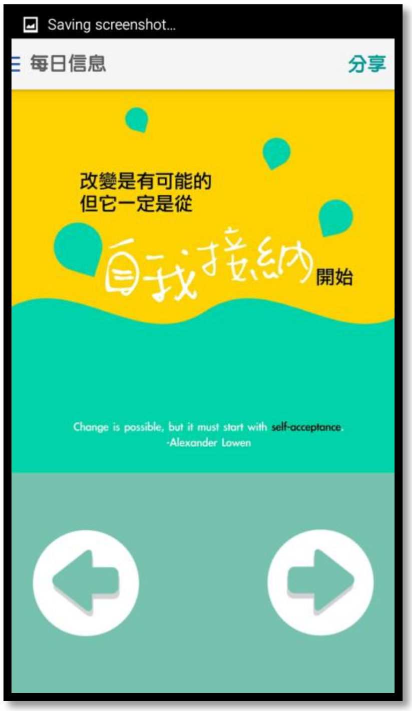

Description: sample well-being tips

Multimedia Appendix 2: Screenshots of the “Living With Heart” Application

Part 4. Screenshot of website layout

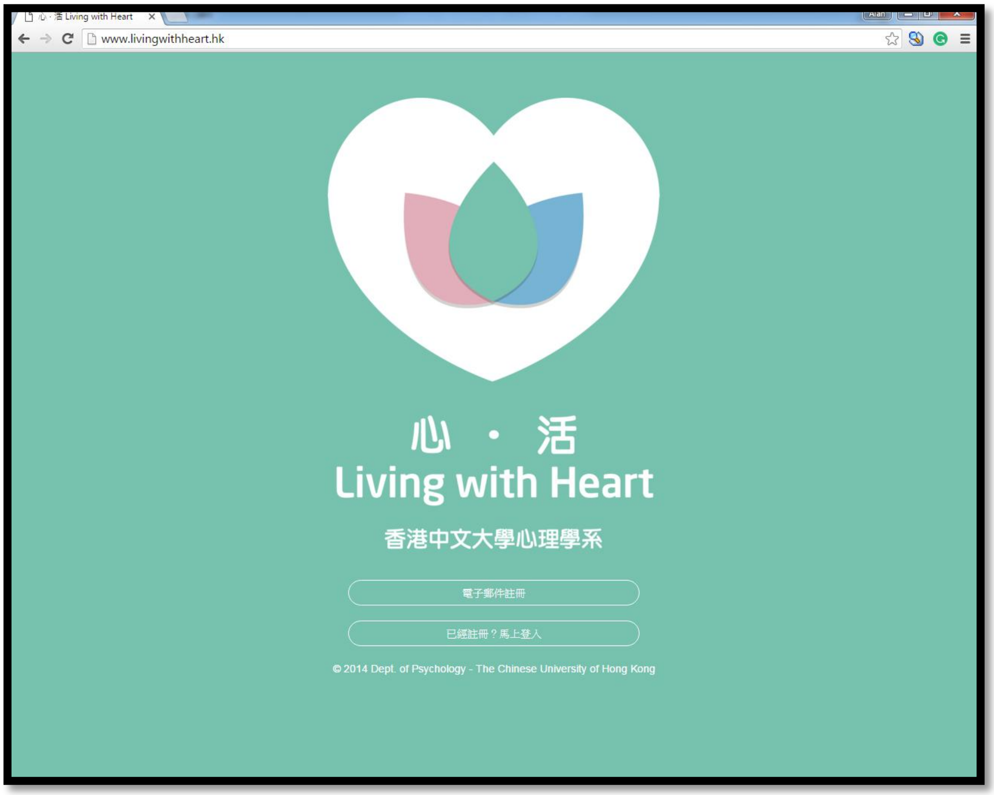

Description: Login page of website

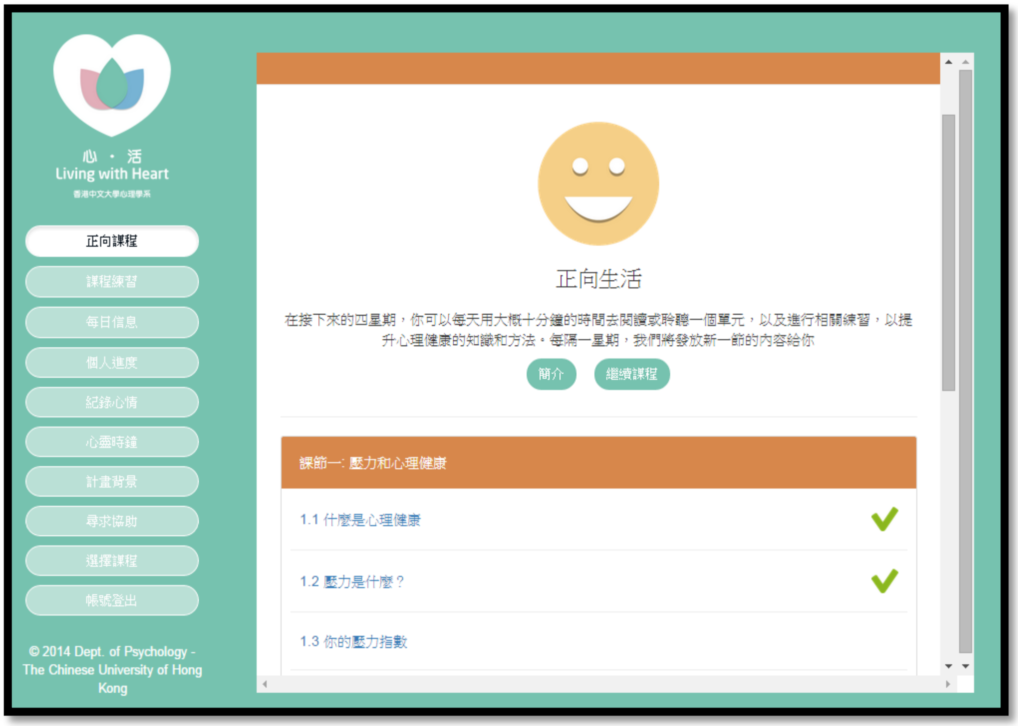

Description: Sample content page
